# Cases of Diphtheria

**Published:** 1860-03

**Authors:** J. J. Morgan

**Affiliations:** Windham, Iowa


### ARTICLE 6.

### CASES OF DIPHTHERIA.

BY J. J. MORGAN, M. D., OF WINDHAM, IOWA.

I was called, October 4th, 1859, to see a child, that was said to be seriously ill with sore throat. I found the child going about the house, disposed to play some. I learned that its illness had been discovered some three days previous to my visit. Upon examination of the throat, I found the tonsils considerably enlarged, and covered with a yellowish white looking substance; a part of which could be detached in the form of small shreds, presenting a dirty bluish color, and only moderately tenacious. The fauces were intensely red, as was the posterior part of the pharynx. The whole of the fauces and throat as far as could be seen, presented a peculiar knotted or lumpy appearance, that I have not observed in any other affection of the throat. The febrile excitement, or heat, was not great, although the pulse was a hundred and ten. I prescribed a saturated solution of acetate of lead, adding one grain of morphine to the ounce; this to be used as a gargle, or applied by means of a sponge to the diseased surface, two or three times during twenty-four hours. I gave chlorate potassa, three grains, once in four hours; quinine in grain doses, twice a day; ordered a saline cathartic, which was not at hand; castor oil was used. Visited patient again two days after, found it apparently much better. Continued treatment same as before. Heard nothing more of the case for six days, when the messenger informed me that the spots (which had almost wholly disappeared) were increasing; and that he wanted another bottle of the gargle. I sent, instead of the lead wash, one of nitrate of silver, thirty grains to the ounce; continued other treatment the same. Was called two days after this, (being fourteen days from the time of my first visit) found the patient greatly reduced; the prostration in a great degree, probably due to a profuse diarrhœa, which had supervened some two days previous. I gave opium and acetate of lead to check the evacuations of the bowels; continued treatment as before. Did not see the case again, which terminated fatally four days after; about twenty-one days from the time of invasion.

In this family, there had been three children buried within six days of my first visit, who were affected, according to the statement of the parent, in the same way as the one that I have just related. They were aged respectively, five, seven and nine. They were treated by an eclectic physician, who also had charge of the case above described, until I was called. Two more of this family were taken in the same manner as the others, aged eleven and thirteen. The oldest, though the tonsils were considerably enlarged, and the fauces and throat highly inflamed, was but mildly affected; but little of the exudation appearing, and the disease subsiding in eight or ten days. But the younger of two was apparently as aggravated as the one that terminated fatally. This case recovered after a protracted illness of three weeks. The only application made to the throat, was the solution of acetate of lead; the other treatment the same as in the first case.

Called October 8th, to Mr. J. M.'s, found a boy four years of age, breathing as with croup. Upon examination, found the tonsils covered with a heavy coating of the false membrane. In the same family, a boy aged twelve years, presented the same symptoms, except the croupal cough, and labored breathing. To the younger of these, who was evidently laboring under croup, I administered an emetic; gave as a wash to the throat, nitrate of silver, thirty grains to the ounce; directed chlorate potassa; as in the other cases, ordered cathartic. The treatment in these two cases was the same, except in the oldest, the lead wash was used, instead of the one of lunar caustic. The younger of the two, the croupal case, continued to improve to all appearance until the second night from my first visit, when it died suddenly as from spasmodic croup—as the mother expressed it, he uttered one shrill whistle and was dead.

At this time two more of this family were attacked, aged seven and nine years. The eldest of these passed rapidly into an aggravated form, the false membrane being extensively developed. I used the lead wash in all these cases some four days, when they appeared to be so much improved, that I discontinued remedies, directing an infusion of oak bark and alum, as a gargle. Ten days after this I was summoned to attend this same family; found the disease present with all its former violence. I here resumed the treatment as before, except the application to the throat, for which I used nitrate of silver in fine powder, applying it by moistening the point of a sponge, to which a sufficient quantity would adhere. I cauterized the whole of the surface that was covered with the exudation, but did not extend it to the inflamed parts that were free from the false membrane. This treatment was continued about four days, when they gradually, but slowly convalesced.

A few words in regard to the fourth and last case in this family; the commencement of which was so mild, as scarcely to attract notice. I simply used the gargle of the solution of lead for a few days. But I learned upon being called on the 16th of November, (about thirty-five days from the first appearance of the affection) that he had not been wholly free from the disease at any time; that the tonsils had remained swollen, and the fauces red and inflamed, but at no time had there been present the characteristic exudation, which was now for the first time extensively developed. In this case the pulverized nitrate of silver was used, continuing it with the general treatment, about six days; when after a duration of some forty days, it could barely be said to be convalescent.— In this case the nitrate of silver was applied for a few days after the false membrane had ceased to be formed.

---

Cholera is said to be prevailing in a very fatal form in the north of Africa. In a body of four hundred artillery-men, one hundred and forty-four died of the cholera in a very short time.

